# Redefining Loyalty: How Political Deviants Maintain Positive Self-Views Amid Ingroup Rejection

**DOI:** 10.3390/bs16010126

**Published:** 2026-01-16

**Authors:** Trystan Loustau, Liane Young

**Affiliations:** Department of Psychology and Neuroscience, Boston College, 275 Beacon St., Chestnut Hill, MA 97331, USA; younglw@bc.edu

**Keywords:** ingroup deviance, group loyalty, group norms, political dissent, partisan identity

## Abstract

Deviance poses a fundamental challenge for groups: while it can threaten cohesion and invite moral condemnation, it can also express deep commitment to shared principles. The present research examines how loyalty shapes perceptions of constructive deviance through the case of Republicans for Harris (RHs) during the 2024 U.S. presidential election. Across three time points, we compared how deviants (RHs, *N* = 89) perceived themselves to how they were viewed by mainstream ingroup members (Republicans for Trump; RTs, *N* = 340) and outgroup members (Democrats; *N* = 294). Results revealed marked asymmetries: RTs viewed RHs as less loyal, less prototypical, and more likely to defect than RHs saw themselves. All groups, including mainstream ingroup members, outgroup members, and deviants themselves, felt warmer toward deviants they perceived as more loyal and prototypical. These findings suggest that constructive deviants maintain positive self-views by construing their actions as expressions of fidelity to, rather than rejection of, the group.

## 1. Introduction

Groups depend on shared norms to maintain cohesion, define boundaries, and pursue collective goals. When members deviate from these norms—by violating expectations, rejecting leadership, or endorsing alternative values—they are often vilified as traitors or dismissed as disengaged from the group (J. Marques et al., 2001; Abrams et al., 2000). Yet research shows that dissent can also emerge from deep commitment to the group itself: highly identified members may challenge norms they perceive as misguided or harmful to the group’s long-term interests (Packer, 2008; Jetten & Hornsey, 2014). When they are cast as disloyal, how do principled dissidents sustain a sense of belonging and moral integrity?

No prior work has directly compared how others evaluate deviants with how deviants perceive themselves. However, examining how perceptions of loyalty shape both perspectives is critical for understanding how deviance is morally justified and psychologically sustained. In the present work, we investigate how deviants interpret their own actions and relationship to the group, and how these self-construals align or diverge from perceptions held by mainstream ingroup members and outgroup members across a period of heightened intergroup conflict. We focus on Republicans for Harris (RHs), Republicans who voted for Democratic candidate Kamala Harris in the 2024 U.S. presidential election, a vivid case of moralized political deviance in an era of extreme polarization.

### 1.1. Evaluations of Deviance

Deviant group members like RHs are often criticized or marginalized because they threaten group cohesion (Abrams et al., 2000), dilute positive distinctiveness from the outgroup (Hornsey, 2016), and impede group goals (Jetten & Hornsey, 2014). Ingroup deviants are most likely to face rejection or disapproval when their actions are seen as selfish (Loustau et al., 2024) or unjustified in the context (Hornsey et al., 2003), when they appear fundamentally irredeemable and misaligned with group values (Abrams et al., 2000), or when the group feels insecure about its standing (J. Marques et al., 2001). Especially in times of uncertainty or intergroup conflict, negative evaluations of ingroup deviants may serve an important group-binding function, reinforcing boundaries of acceptable behavior and establishing norms around conformity to help maintain group cohesion (Stephan et al., 2015).

Evaluations of dissidents are fundamentally shaped by loyalty, a moralized commitment to uphold and protect the ingroup (Berry et al., 2021). We distinguish loyalty as (1) a trait-level moral virtue, reflecting a stable disposition to prioritize and defend one’s group (Levine & Moreland, 2002; Graham et al., 2013); (2) a set of group-supporting behaviors, encompassing concrete acts that affirm solidarity and collective welfare (Jetten et al., 2003); and (3) prototypicality, the degree to which an individual exemplifies group-defining norms, values, and identity (Turner et al., 1987)—that is, the subjective “fit” of an individual within a group, including shared qualities and an understanding of group expectations. Prior work demonstrates that the more group members are perceived as loyal, expected to support the group in the future, and seen as prototypical, the more positively they are evaluated (J. Marques et al., 2001; Turner et al., 1987; Jetten & Hornsey, 2014).

However, it remains unclear how these factors shape dissident group members’ self-perceptions. Prior research has largely focused on the motivations for dissent, such as beliefs about the feasibility of change, anticipated long-term benefits to the group, and concern for its overall vitality (Packer & Miners, 2014; Dupuis et al., 2016), but less on how deviants understand their own loyalty and how these perceptions inform their self-evaluations. One possibility is that dissidents devalue loyalty, construing their deviance as principled disloyalty, a willingness to challenge the group for moral or pragmatic reasons. Alternatively, they may interpret their behavior as loyal dissent, rooted in the belief that genuine commitment sometimes requires confronting misguided or harmful group norms. Framing their actions as expressions of loyalty may enable deviants to reconcile their dissidence with their group identities.

To the extent that deviants may value loyalty, they may still differ from mainstream members in whom they feel compelled to demonstrate loyalty toward. Prior work suggests that when loyalty to one’s group conflicts with broader moral obligations, individuals prioritize whichever loyalty feels most salient (Dungan et al., 2019). In the case of Republicans for Harris, their deviance was often framed as prioritizing country over party; that is, maintaining loyalty to the well-being of Americans generally over the electoral success of the Republican Party. For instance, several Republican leaders who endorsed Harris explicitly justified their deviance as preserving broader American values, such as democracy and decency, which they viewed as threatened under Donald Trump’s leadership (e.g., NPR, 2024). To test this possibility, we also examined the extent to which deviants and others perceived RHs as prioritizing country over party.

Finally, previous work shows that the strength of one’s group identity can shape attitudes toward deviance. Research on the Black Sheep Effect demonstrates that high identifiers often react more harshly to ingroup deviants because such members threaten the group’s image and cohesion (J. M. Marques et al., 1988; Abrams et al., 2000). At the same time, research on principled deviance suggests that strong identifiers may also be more likely to dissent when they perceive the group as violating its core values or moral standards (Packer, 2008; Jetten & Hornsey, 2014). Thus, group identification can both suppress and motivate deviance depending on whether loyalty is expressed through conformity or reform. Importantly, identification with the outgroup may also play a distinct role. Group members who are more open to the outgroup may place less importance on rigid norms that emphasize the positive distinctiveness of their group relative to others, thereby judging deviance less negatively.

### 1.2. The Present Case: Republicans for Harris

The case of Republicans for Harris offers a uniquely revealing lens through which to study perceptions and evaluations of deviance for several reasons. First, deviance within political groups is especially costly in the current era of social sorting and polarization. With deepening polarization (ANES, 2020), partisans increasingly perceive both in-party and out-party norms in extreme and stereotypical terms (Fernbach & Van Boven, 2022; Ahler, 2018). At the same time, other social identities have become more tightly aligned with political affiliation (Lelkes, 2018; Mason & Wronski, 2018), heightening conformity pressures as individuals seek social acceptance and moral certainty within their political communities (Turner, 1991). Party members are thus motivated to embody the traits of a prototypical partisan (Packer et al., 2021; Luttig, 2016), particularly during moments of heightened intergroup conflict. Despite these strong incentives for conformity, a coalition of “Republicans for Harris” emerged across states and social media in 2024, led in part by hundreds of former Republican staffers who publicly endorsed Vice President Kamala Harris (NPR, 2024). Exit polls suggested that approximately five percent of Republicans (approximately three million voters) supported the Democratic ticket (Molski, 2024). This cross-party defection represents an increasingly rare and socially costly form of deviance, providing an ideal context to examine how loyalty, identity, and moral conviction shape both deviant actors’ self-perceptions and others’ evaluations of them.

Second, the salience of intergroup threat surrounding the election period further amplified the meaning of this deviance. In periods of intense partisan conflict, acts of defection carry symbolic weight: they can signal an ingroup’s vulnerability and lack of cohesion while simultaneously strengthening the outgroup’s relative position (J. M. Marques et al., 1988 Abrams et al., 2002). To investigate how the salience of intergroup conflict shapes evaluations of deviance, we assessed perceptions of RHs across multiple time points before and after the election. This temporal design allowed us to capture how the evolving political climate influenced judgments of loyalty, prototypicality, and moral character.

Finally, the election context provided a fitting opportunity to examine outgroup members’ perceptions of dissidents. The Harris campaign’s explicit appeals to anti-Trump Republicans (McCammon, 2024) suggest that Democrats may have evaluated RHs more positively, as allies in an intergroup struggle. Although prior work shows that people sometimes admire outgroup members who defect from rival groups (Frimer & Skitka, 2018) or use such deviance to justify outgroup derogation (Van Assche et al., 2020), little is known about how outgroup members perceive the loyalty of such dissidents, or how these perceptions shape broader attitudes toward deviance. Individuals may praise disloyalty among outgroup members when it benefits their own group, but they may also disparage outgroup dissidents as untrustworthy. In the current study, we therefore examined both mainstream ingroup members’ and outgroup members’ perceptions and evaluations of deviants across time.

### 1.3. Current Study

The present study examines how deviant group members, Republicans for Harris (RHs), were perceived and evaluated by mainstream ingroup members (Republicans for Trump), outgroup members (Democrats), and themselves. Across three time points surrounding the election, we assess feelings of warmth toward RHs. We focus on how these attitudes are shaped by perceptions of loyalty, conceptualized as a trait-level moral virtue, a set of group-supporting behaviors, prototypicality, and the prioritization of competing norms (i.e., “country over party”).

Whereas prior research has primarily focused on how perceived loyalty shapes others’ judgments of deviants, we extend this work by examining how it shapes dissidents’ own construals of their actions, revealing how deviants sustain positive self-evaluations despite harsh reactions from mainstream ingroup members. We also investigate how identification with both the ingroup and outgroup influences evaluations of deviance. Together, these studies examine the extent to which perceptions of loyalty underlie both self-evaluations and others’ evaluations of deviance, and how individuals may differ in how they define and express loyalty.

All preregistrations, materials, data, analysis scripts, and Supplementary Materials for the current studies are available on OSF: https://osf.io/7t9mw/?view_only=e2c602b8fe514d69be6e01428f855978 (accessed on 7 January 2026).

## 2. Methods

### 2.1. Participants

Data were collected at three time points: September 2024 (“6 wks pre”), 29 October–5 November (“1 wk pre”), and 9 November–23 November (“2 wks post”). To identify Republicans for Harris, we conducted a pre-survey on Prolific of 3000 self-identified Republicans and asked them to report both their partisan affiliation and 2024 U.S. presidential candidate preference. Those who selected “Republican” and “Kamala Harris” were invited to participate in our longitudinal study. To maximize inclusion, participants were allowed to enter at any wave, enabling us to maximize the number of Republicans for Harris (RH) included. Of the 122 identified RH participants, 89 completed Time 1, and the final sample included 92 unique individuals across the three waves. Thirty-two participants (36%) completed all three waves, while 29 (33%) completed two waves, and 34 (38%) completed only one wave. This partially overlapping longitudinal design allowed us to retain a core subsample for within-person analyses while increasing between-subjects power.

#### Attrition

Participant retention was high overall, though it varied somewhat across groups and waves (see Table 1). Of the 723 participants who completed the Time 1 survey (six weeks before the election), 489 (67.6%) completed the Time 2 survey (one week before the election), and 450 (62.2%) completed the Time 3 survey (two weeks after the election). Attrition from Time 1 to Time 2 ranged from 24% to 42% across groups, while attrition from Time 2 to Time 3 was lower, ranging from 5% to 16%. Attrition was highest among Republicans for Harris, likely because this subgroup is comparatively rare in both the U.S. population and on Prolific, resulting in a smaller and less stable recontact pool. Additionally, niche subgroups tend to be less active on Prolific, reducing recontact rates and increasing missed invitations. Mean age and gender composition remained stable across waves, suggesting that dropout did not systematically alter the demographic makeup of the sample. Sample demographics are summarized in Table 1. All Republicans for Harris and Democrat participants indicated a candidate preference for Kamala Harris at all three time points. All Republicans for Trump indicated a preference for Donald Trump at all three time points.

### 2.2. Measures

#### 2.2.1. Warmth

Participants rated how warmly or coldly they feel toward RHs, RTs, and Democrats on a slider scale from −100 (very cold) to 100 (very warm).

As preregistered, we conducted an exploratory factor analysis (EFA) on perception items collected at Time 1, six weeks before the election. Three distinct factors emerged: Republican Loyalty, Perceived Likelihood of Supporting the Republican Party, and Perceived Likelihood of Dissenting from the Republican Party. Items that loaded highly on each factor were averaged to create composite indices at each time point. One item did not load strongly on any factor and was therefore examined independently (Perceived Norm Priority). Full EFA results are provided in the Supplementary Materials.

#### 2.2.2. Republican Loyalty

Participants rated the extent to which they agreed or disagreed with three statements: “[RTs/RHs] are traitors”, “[RTs/RHs] have betrayed their party”, and “[RTs/RHs] are TRUE Republicans” on a 6-pt scale from “Completely disagree” to “Completely agree”. The first two items were reverse-coded. We observed high internal consistency across items for both target groups (RH: α = 0.882, RT: α = 0.904).

#### 2.2.3. Perceived Norm Priority (Country over Party)

Participants rated the extent to which they agreed or disagreed with the statement “[RTs/RHs] are prioritizing country over party” on the 6-point scale.

Intentions Toward the Group. To assess the perceived likelihood of supporting the Republican Party, participants indicated the extent to which they agreed or disagreed with three statements: “[RTs/RHs] are likely to vote in favor of conservative policies in the future”, “[RTs/RHs] are likely to support Republican candidates in other elections”, and “[RTs/RHs] likely have many friends who are Republican” on the 6-point scale. We observed high internal consistency across items for both target groups (RH: α = 0.828, RT: α = 0.763). To assess the perceived likelihood of dissenting from the Republican Party, participants indicated the extent to which they agreed or disagreed with four statements: “[RTs/RHs] are likely to vote in favor of liberal policies in the future”, “[RTs/RHs] are likely to support Democratic candidates in other elections”, “likely have many friends who are Democrat”, and “are likely to become Democrat in the future” on the 6-point scale. We observed high internal consistency across items for both target groups (RH: α = 0.841, RT: α = 0.843).

#### 2.2.4. Prototypicality

We also aimed to assess how closely participants associated targets with each political party by rating their impressions of how Republican (“Republican-ness”) and Democrat (“Democratic-ness”) they perceive RHs, RTs, and Democrats to be on a slider scale from 0 (not at all) to 100 (extremely).

#### 2.2.5. Partisan Identification

Participants rated how strongly they identify with Republicans and Democrats, separately, on 7-pt scales from 1 (not at all) to 7 (extremely strongly).

#### 2.2.6. Candidate Support

Participants indicated which presidential candidate they supported for the 2024 presidential election. At the second (1 wk pre) and third (2 wks post) time points, participants were asked if they had voted, and, if so, which candidate they voted for.^1^

### 2.3. Analysis Approach

Although our preregistered analytic plan specified ANOVAs and *t*-tests, we ultimately employed regression and mixed-effects models to account for the partially overlapping longitudinal design. This approach retained all available data and appropriately modeled repeated measures while accounting for individual-level variability. To examine predictors of attitudes across time, we fit a series of linear mixed-effects models (LMEs) including perceived norm priority, perceived party loyalty, perceived likelihood of party support, perceived likelihood of party dissent, perceived Republican-ness, perceived Democratic-ness, and personal identification with Republicans and Democrats as fixed effects. Participant was included as a random intercept, and parameters were estimated via (restricted) maximum likelihood. Analyses used all available observations per model; observations with missing values on model variables were excluded listwise. Degrees of freedom were estimated using the Satterthwaite approximation. We fit separate models for each sample (Republicans for Trump, Republicans for Harris, and Democrats) paired with each target (Republicans for Trump, Republicans for Harris), yielding six total models. Additionally, to evaluate the influence of each predictor at each time point, we conducted separate multiple linear regressions for each wave. Model summaries appear in Table 2, and full results are available in the Supplementary Materials.

### 2.4. Post Hoc Sensitivity Analyses

Post hoc sensitivity analyses based on Wald z-tests estimated the minimum detectable effect sizes (MDES) for each fixed effect in the mixed-effects models predicting warmth. The model predicting warmth toward RHs among Republicans for Trump (RTs) had 80% power to detect effects between approximately β = 3.3 and β = 5.1. Observed effects for trait-based loyalty, expected future Republican support, Republican-ness, and Democratic identification exceeded this threshold. The model predicting warmth toward RHs among Democrats had 80% power to detect effects between approximately β = 3.6 and β = 5.7. Observed effects for trait-based loyalty, expected future dissent, Democratic-ness, and Democratic identification exceeded this threshold. Finally, the model predicting warmth toward RHs among Republicans for Harris (RHs) had 80% power to detect effects between approximately β = 6.0 and β = 8.1, with only the effect of trait-based loyalty exceeding this range. Across models, the remaining predictors did not exceed their respective MDES thresholds, indicating that smaller effects were likely underpowered.

## 3. Results

**Figure 1:**
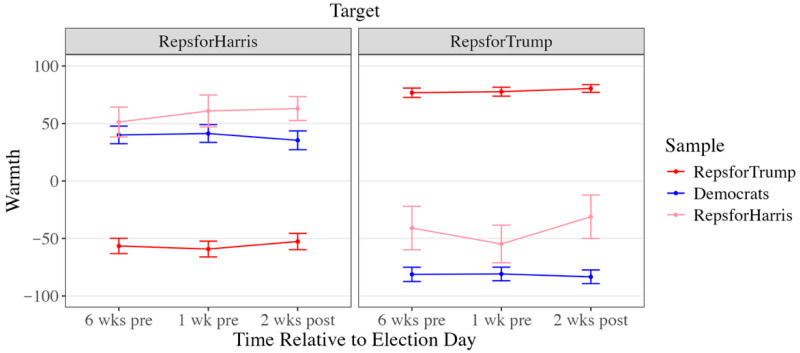
Warmth Toward Target by Sample. Note. Error bars represent 95% confidence intervals.

**Figure 2:**
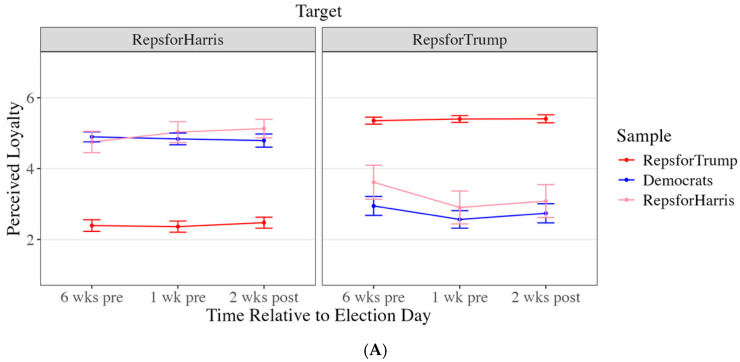

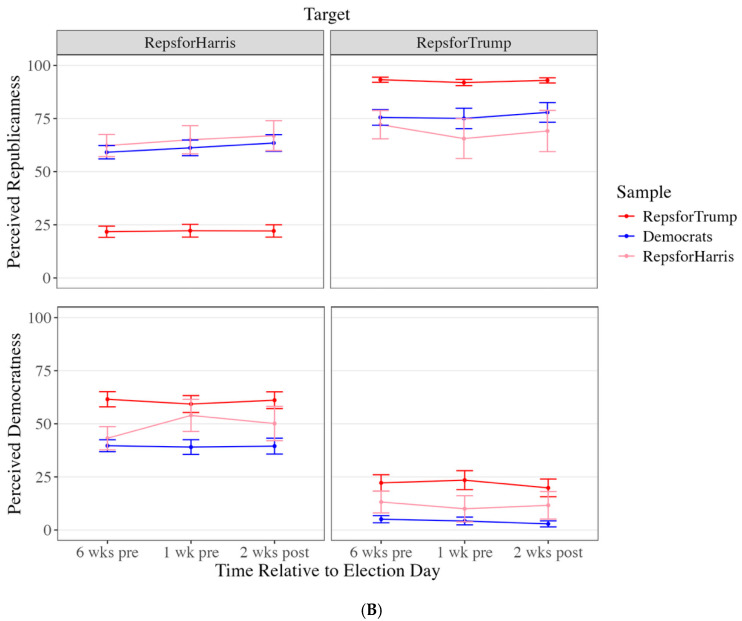
(**A**) Perceived Trait Loyalty of Target by Sample. (**B**) Perceived Group Prototypicality Per Target by Sample. Note. Error bars represent 95% confidence intervals.

**Figure 3:**
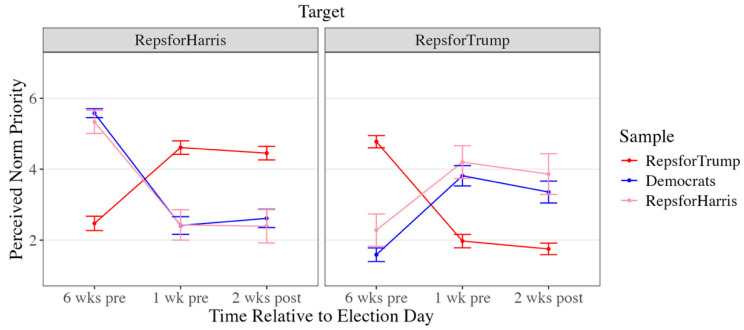
Perceived Norm Priority (Country Over Party) Per Target by Sample. Note. Error bars represent 95% confidence intervals.

Results are organized around three questions. First, how are deviants affectively evaluated by mainstream group members and other deviants (Figure 1)? Second, how loyal are deviants perceived to be (Figure 2), and do those perceptions predict warmth (Table 2)? Third, does “country over party” function as a stable moral justification, or does its meaning shift across the election period (Figure 3)?

### 3.1. Warmth Toward Deviants

Across time, clear asymmetries emerged in affective evaluations of mainstream and dissident group members. On average, mainstream Republicans (RTs)’ warmth toward dissident Republicans (RHs) was significantly below zero but significantly greater than zero for fellow mainstream Republicans. As shown in Figure 1, RTs felt no warmer toward RHs than toward Democrats, *b* = −2.99, *t*(2143) = −1.57, *p* = 0.261, 95% CI [−7.48, 1.49], indicating that dissident ingroup members were viewed as negatively as outgroup members. Whereas prior work finds that group members are often more receptive to criticism of their group when it originates from ingroup members compared to outgroup members because it is less threatening to their social identities (i.e., the intergroup sensitivity effect; Reiman & Killoran, 2023), these results suggest that mainstream Republicans may have felt highly threatened by both dissident inparty members and outparty members during this period.

Conversely, dissident Republicans (RHs)’ warmth toward mainstream Republicans was significantly below zero, but significantly above zero for fellow dissidents. RHs felt warmer toward Democrats than toward mainstream Republicans, *b* = 77.00, *t*(453) = 17.11, *p* < 0.001, 95% CI [66.40, 87.50], and warmer still toward fellow dissidents, *b* = 30.50, *t*(453) = 6.78, *p* < 0.001, 95% CI [19.90, 41.10], suggesting that dissident ingroup members felt considerable antipathy toward mainstream ingroup members.

Similar to RHs, Democrats’ warmth toward mainstream Republicans was significantly below zero, but significantly above zero for dissident Republicans (RHs). However, Democrats still felt less warm toward RHs than toward fellow Democrats, *b* = −28.00, *t*(1610) = −5.11, *p* < 0.001, 95% CI [−32.40, −23.70], indicating that while Democrats were relatively more favorable toward outgroup dissenters than toward mainstream outgroup members, ingroup favoritism persisted.

### 3.2. Perceived Loyalty of Deviants

As shown in Figure 2A, RHs rated themselves as more loyal, *b* = 2.26, *t*(991) = 20.79, *p* < 0.001, 95% CI [2.01, 2.52], more likely to support the Republican Party, *b* = 1.18, *t*(971) = 11.62, *p* < 0.001, 95% CI [0.94, 1.42], and less likely to dissent, *b* = −13.34, *t*(879) = −4.84, *p* < 0.001, 95% CI −19.81, −6.86], than RTs perceived them to be, suggesting that deviants viewed their defection as an expression of loyalty and ongoing alignment with party goals, rather than as acts of defection or betrayal. Additionally, RHs and Democrats viewed RHs as more Republican than RTs perceived them to be (RHs: *b* = 40.81, *t*(882) = 16.55, *p* < 0.001, 95% CI [34.90, 46.48]; Democrats: *b* = 37.27, *t*(730) = 22.59, *p* < 0.001, 95% CI [33.33, 41.07]) and less Democratic than RTs perceived them to be (RHs: *b* = −13.34, *t*(879) = −4.84, *p* < 0.001, 95% CI [−19.81, −6.86]; Democrats: *b* = −20.79, *t*(749) = −11.38, *p* < 0.001, 95% CI [−25.08, −16.50]), yet as moderately high on both traits (Figure 2B). These results suggest that ingroup deviants and outgroup members perceived deviants as straddling partisan boundaries, embodying qualities of both parties. This duality further reinforces evaluations of their dissent as principled and grounded in party loyalty.

### 3.3. How Perceived Loyalty Shapes Warmth

Across time, perceiving group members as more loyal to their party was consistently associated with greater warmth toward them. This pattern held across samples and for both mainstream Republicans (RTs) and dissident Republicans (RHs), indicating that even group deviants valued faithfulness to one’s party. These results suggest that deviants and mainstream group members feel negatively toward each other for similar reasons: they perceive the other as traitors to the group. Democrats also expressed warmer attitudes toward both mainstream and dissident Republicans, whom they saw as more loyal to the Republican Party. Extending prior work suggesting that loyalty is a fundamental moral value (Graham et al., 2011), this work suggests that loyalty can be respected even among outgroup members.

In addition to perceptions of deviants’ loyalty and adherence to privileged group norms, evaluations of group deviants are also shaped by expectations about their future behavior toward the group (Jetten et al., 2003). In line with this work, mainstream Republicans (RTs) who perceived dissident Republicans (RHs) as more likely to support the Republican Party felt warmer toward them, suggesting that expectations of future alignment with party goals buffered against negative reactions to deviance. Perceived likelihood of future dissent was not significantly associated with RTs’ warmth toward RHs, indicating that anticipating future dissent did not predict attitudes toward deviants above and beyond the effects of the other predictors.

The extent to which RHs perceived themselves as likely to support or dissent from the Republican Party was not significantly associated with their attitudes toward themselves. However, these null effects should be interpreted with caution, as they may reflect limited statistical power rather than the absence of a meaningful relationship between expected future behaviors and deviants’ self-evaluations. In contrast to mainstream Republicans, Democrats felt warmer toward RHs they expected to dissent from the Republican Party in the future, suggesting that although Democrats may view party loyalty as an admirable trait in the abstract, they respond more positively to outgroup deviants whose anticipated dissent is seen as advancing their own group’s interests.

Attitudes toward deviant group members may also be shaped by how prototypical or representative they perceive those group members to be. When deviance is viewed as more common or normative, it may be less likely to be derogated. In line with this, mainstream Republicans who perceived RHs as higher in Republican-ness (i.e., more Republican) reported warmer attitudes toward them. This effect was consistent at each time point, suggesting that mainstream Republicans evaluated deviants more positively when they viewed them as more prototypical group members. Similarly, RHs and Democrats who perceived RHs as more Republican also reported feeling warmer toward them. At the same time, both groups felt warmer toward RHs they saw as more Democratic.

Finally, we examined whether participants’ own partisan identification influenced evaluations of deviance. Contrary to work suggesting that stronger identification is associated with harsher reactions to ingroup deviants (Reiman & Killoran, 2023), identification with Republicans was not significantly associated with warmth toward Republican deviants. Instead, identification with Democrats was consistently related to warmer attitudes toward deviants. These results suggest that mainstream and dissident Republicans who are more open to outparty members may place less importance on rigid conformity to party norms, thereby reducing negative evaluations of dissident group members. Likewise, Democrats with stronger partisan identities may be particularly inclined to praise outparty deviance when it serves their party’s interests. Extending work showing that affective polarization is driven more by outparty animosity than inparty favoritism (Amira et al., 2021), these findings indicate that evaluations of deviant political group members may hinge less on partisan identity strength and more on broader intergroup openness and willingness to recognize shared moral ground.

#### Context-Dependent Norm Prioritization

Although all participants appeared to value loyalty to one’s group, they diverged in who they perceived as loyal (mainstream members or dissidents). These differing perceptions suggest participants may have construed loyalty differently. To examine this possibility, we assess how perceived norm priority—that is, whether dissident group members were seen as prioritizing one norm (loyalty to country) over another (loyalty to party)—shaped warmth toward deviants. The influence of perceived norm priority on attitudes toward dissidents fluctuated across time points, indicating that expectations for how norms should be prioritized are context-dependent, rather than fixed.

Initially (six weeks before the election), both mainstream and dissident Republicans who perceived dissidents as prioritizing country over party felt warmer toward them, suggesting that putting the country’s welfare first was construed as a prosocial ideal, a sign of moral integrity and concern for the collective good. As shown in Figure 3, both RHs and RTs perceived themselves as more likely than the other to be prioritizing country over party, seeing their own stance as reflecting concern for the best interests of all Americans (RHs: *b* = 2.99, *t*(280) = 14.60, *p* < 0.001, 95% CI [2.59, 3.39]; RTs: *b* = 2.17, *t*(1666) = 20.26, *p* < 0.001, 95% CI 1.96, 2.38]).

However, one week before the election, this relationship flipped: mainstream and dissident Republicans who perceived RHs as prioritizing country over party reported feeling less warm toward them. As the election drew near and intergroup threat intensified, the same “country over party” stance appeared to be recast as condemnable disloyalty to party ideals and long-term success. This shift paralleled changes in perceived norm priority: whereas earlier in the cycle RHs and RTs saw themselves as more likely than the other to prioritize country over party, by this point each group saw itself as less likely to do so (RHs: *b* = −1.68, *t*(280) = −6.15, *p* < 0.001, 95% CI [−2.22, −1.14]; RTs: *b* = −2.60, *t*(1666) = −21.02, *p* < 0.001, 95% CI [−2.85, −2.36]). Although they differed in how they construed what it meant to prioritize party (i.e., supporting Trump vs. Harris), both ultimately placed party loyalty ahead of country in the high-stakes pre-election context.

After the election and the threat of interparty conflict was reduced, there was no significant effect of perceived norm priority on warmth toward deviants, further suggesting that context shifted participants’ perceptions of group norms.

## 4. Discussion

The present study offers a nuanced examination of the cognitive and moral factors that shape attitudes toward deviants, specifically Republicans for Harris (RHs), as perceived by mainstream ingroup members (Republicans for Trump; RTs), outgroup members (Democrats), and the deviants themselves. Across the election period, mainstream ingroup members evaluated deviants as negatively as they did outgroup members, whereas both deviants and outgroup members evaluated deviants more positively than mainstream ingroup members did. Despite stark differences in how loyal they perceived deviants to be, both deviants and mainstream ingroup members valued loyalty, suggesting that constructive deviants like RHs reconcile their deviance with positive group identities not by devaluing loyalty and conformity to group norms, but by construing loyalty in ways that align dissent with the group’s deeper principles.

### 4.1. How Loyalty Shapes Attitudes Toward Deviance

In the current study, we distinguished among multiple dimensions of loyalty to better understand how perceived loyalty shapes evaluations of deviance. We examined trait-based loyalty, the extent to which deviants were viewed as having betrayed or remained faithful to their group; action-based loyalty, the extent to which they were expected to support or dissent from the group in the future; and representational loyalty, the extent to which they were perceived as prototypical group members who embody the group’s norms and values.

Clear asymmetries emerged between dissidents’ self-perceptions and mainstream ingroup members’ evaluations. Deviants rated themselves as more loyal, more likely to support the group in the future, less likely to challenge it, and more prototypical than mainstream members perceived them to be. These findings extend prior work suggesting that deviance can arise from strong identification with the group rather than disengagement (Packer, 2008) by showing that constructive deviants view themselves as deeply committed to the group, in terms of both their sense of alignment with group norms and future intentions.

Further building on this work, we find that perceived loyalty was closely associated with evaluations of ingroup members, not only among mainstream ingroup members but also among deviants themselves. Across time, RTs expressed greater warmth toward both RHs and fellow RTs they perceived as more loyal, consistent with prior work showing that reactions to deviance function as social signals that reinforce group conformity (Cramwinckel et al., 2015). These reactions appeared calibrated to the perceived degree of disloyalty, with stronger disapproval directed at those seen as most traitorous. Importantly, RHs exhibited the same pattern, reporting greater warmth toward both RHs and RTs they viewed as more loyal. Together, these findings indicate that deviants maintain positive self-views not by devaluing loyalty and adherence to group norms, but by construing themselves, similar to mainstream ingroup members, as faithful and committed to their community.

Strikingly, even Democrats reported more positive attitudes toward both RHs and RTs they saw as more loyal, suggesting that trait-level loyalty may serve as a cross-cutting moral virtue that elicits respect even across partisan divides. For outgroup members, perceived loyalty may be particularly important in shaping impressions of deviants, who might otherwise appear opportunistic or untrustworthy when their dissent aligns with outgroup goals. In this way, perceived trait loyalty helps buffer against negative or suspicious reactions among outgroup observers.

While perceived trait-based loyalty was consistently associated with warmer attitudes, the influence of expected future loyalty varied across groups. Mainstream ingroup members felt warmer toward deviants when they expected them to support the group in the future. This pattern can be interpreted in two related ways: mainstream ingroup members may form positive attitudes toward deviants when they construe the lack of support as an isolated instance rather than a broader pattern, or as a form of dissent tied specifically to one norm (i.e., the rejection of Trump) rather than to group norms more broadly. In either case, mainstream ingroup members who viewed the deviants’ actions as limited in scope, rather than as a sign of enduring lack of support for the group, evaluated them more positively.

By contrast, expected future dissent (i.e., anticipated support for the outgroup) did not significantly influence mainstream ingroup members’ attitudes toward deviants, indicating that perceived alignment with the opposing side did not predict warmth beyond perceived alignment with the ingroup. In this way, mainstream ingroup members appear to have valued loyalty primarily as an affirmation of ingroup commitment rather than as an active rejection of the outgroup. Consistent with this interpretation, mainstream ingroup members felt warmer toward deviants they perceived as more prototypical of the ingroup, whereas perceived outgroup prototypicality showed no reliable association with warmth.

Similarly, Democrats appeared to place greater importance on deviants’ alignment with their own group than with the outgroup (i.e., Republicans). They expressed greater warmth toward RHs they expected to exhibit future dissent (i.e., support for the Democratic Party), but their evaluations were not significantly associated with expected future support for the Republican Party. This asymmetry may reflect differences in how clearly group norms are defined: what it means to be loyal to the ingroup is likely more salient and concretely prescribed than what it means to be loyal to the outgroup. Consequently, people may base their evaluations of deviants more on expected conformity to markers of ingroup loyalty than on the extent to which deviants distance themselves from the opposing side.

Among deviants themselves, expectations of future support or dissent were not significantly tied to self-evaluations, suggesting that prospective, action-based expressions of loyalty may play a less central role in how deviants construe their own group commitment. This pattern may reflect a difference in the relative importance of behavioral loyalty for deviants compared to mainstream group members, though further research is needed to clarify this distinction. Even so, similar to mainstream ingroup members, deviants who perceived themselves as more prototypical group members evaluated themselves more positively. This finding suggests that constructive deviants do not maintain a positive self-image by deemphasizing the importance of adhering to group norms broadly but rather by defining group norms in ways that align with their own understanding of the group’s core values. In line with this, deviants who saw themselves as more prototypical of the outgroup also reported more positive self-evaluations, suggesting that constructive deviants may reframe group boundaries to include values or norms shared with the outgroup, thereby maintaining self-consistency while rejecting aspects of the group they view as misguided.

Likewise, outgroup members also felt warmer toward deviants when they viewed them as more prototypical of both the ingroup and outgroup. Seeing deviants as characteristic of their own group may alleviate concerns about inconsistency or untrustworthiness, whereas seeing them as characteristic of the outgroup may highlight shared moral principles or common ground.

### 4.2. Context-Dependent Construals of Loyalty

In the current work, we examined whether deviants evaluate themselves more positively than mainstream ingroup members because they prioritize different norms than mainstream ingroup members: specifically, loyalty to country over loyalty to party. However, we found that deviants’ norm priorities closely mirrored those of mainstream ingroup members over time. Both groups valued prioritizing country over party six weeks before the election but favored party over country one week before the election, suggesting that the evolving election context similarly shaped their moral priorities. Early in the election cycle, before intergroup threat was most salient, loyalty to national well-being may have been regarded as a prosocial ideal. As the election approached and partisan threat intensified, this same stance was reframed as condemnable disloyalty, reversing its effect on attitudes.

These results further suggest that, similar to mainstream ingroup members, constructive deviants continue to prioritize loyalty to the group but likely differ in how they interpret what that loyalty entails. This pattern echoes classic work showing that political deviants actively seek or construct protective environments that allow them to maintain positive self-concepts despite normative opposition (Finifter, 1974). Although we did not directly assess how RHs construed normative conflicts within their group, it is possible that they distinguished between loyalty to Trump and loyalty to the Republican Party. As Republican identity has increasingly become organized around allegiance to Trump (M. J. Lee, 2017), dissenters may have perceived their stance as consistent with loyalty to the party’s underlying values, even while rejecting its current leader. Other Republican dissenters have similarly justified progressive positions—such as support for abortion rights (C. Lee, 2024), same-sex marriage (Schnell, 2022), and climate action (Groves, 2023)—by appealing to conservative principles like personal freedom, economic growth, and national security.

### 4.3. Limitations

Several limitations warrant consideration. First, our sample of Republicans for Harris was limited. RHs represent a rare and hard-to-reach subgroup, comprising only a small fraction of the Republican electorate. Although our targeted sampling strategy enabled us to recruit a meaningful number of RHs, the resulting sample was modest (N = 89) and declined over time, limiting statistical power for within-person analyses. Nonetheless, this study is the first to directly compare real-world deviants’ self-perceptions with how they are perceived by both ingroup and outgroup members. As such, it provides a rare and ecologically valid case through which to examine how deviance is experienced and evaluated in a polarized political context. As a robustness check of these results, we conducted an exploratory analysis of political attitudes among mainstream and deviant political group members using the American National Election Studies 2024 Time Series Study (ANES, 2025; see Supplementary Materials). Since this study did not include a measure of attitudes toward deviants, we instead examined evaluations of the presidential candidates. Consistent with our warmth results, RHs reported less warmth toward Harris than Democrats but more than RTs, and more warmth toward Trump than Democrats but less than RTs. These patterns provide converging evidence that political deviants demonstrate distinct evaluations of mainstream group members; however, they do not speak to how deviants themselves are evaluated. Thus, future work is needed to further examine the factors that shape evaluations of deviants.

Second, our longitudinal data collection window was limited. Although we collected data across three time points surrounding the 2024 election, perceptions of RHs have likely continued to evolve after the final time point (two weeks following the election). Longitudinal research over a longer period with larger samples of ingroup deviants could further illuminate how perceptions of deviants and their self-perceptions change over time, and in response to shifts in group status uncertainties.

Third, our focus on Republican deviants limits the generalizability of our findings to Democrat deviants and non-political groups. Prior work suggests that conservatives tend to place greater emphasis on conformity and the moral foundations of loyalty and authority (Graham et al., 2009; Jost et al., 2018), potentially leading to harsher judgments of deviance that threaten group cohesion or leadership. However, deviance derogation occurs across the political spectrum (Kulibert et al., 2025), and both liberals and conservatives endorse obedience to authority figures aligned with their own ideology (Frimer et al., 2014). Consistent with this, both mainstream Republicans and Democrats in our study felt warmer toward deviants they viewed as more loyal to and prototypical of the Republican Party, suggesting that Democrats may likewise value loyalty and derogate ingroup members they perceive as disloyal. Additionally, in our supplemental analysis of political deviants using data from the ANES 2024 Time Series Study, we examined political deviants among Democrats (i.e., Democrats who supported Trump), and found that, similar to Republicans who supported Harris, these individuals exhibited reduced inparty favoritism and outparty antipathy in their evaluations of Harris and Trump. Although these findings do not speak to how Democratic deviants are evaluated by others, they point to notable similarities in deviants’ own intergroup evaluations across partisan groups.

Since partisanship is especially polarized and morally charged (Garrett & Bankert, 2020; Loustau et al., 2025), it provided a robust context for examining how deviants maintain positive self-views when the social costs of dissent are so high. In non-political domains, in which the costs of deviance may be lower, deviants may face less social sanction and thus have greater flexibility to reconcile dissent with group commitment. Future research should examine whether Democrats’ and non-political groups’ responses to deviance follow similar psychological mechanisms or diverge in ways that reflect distinct moral priorities.

## 5. Conclusions

This study offers a nuanced analysis of deviance during a period of heightened intergroup conflict, focusing on Republicans for Harris during the 2024 U.S. presidential election. The findings suggest that constructive deviants interpret their actions not as disloyalty, but as an alternative way of demonstrating fidelity to the group’s core principles. Like mainstream ingroup members, they anchor their positive self-evaluations in perceptions of loyalty and prototypicality, viewing themselves as committed members who embody the group’s true values. This work reveals how dissent can coexist with loyalty, illustrating that moral disagreement within groups may stem less from differences in how much loyalty is valued than from competing understandings of what it means to be loyal.

## Figures and Tables

**Table 1:** Sample Statistics Across Studies.

**Time 1—6 Wks Pre**						
	**N_Total_**	**Attrition**	**Age**	**Gender**		
			**M (SD)**	**N_Female_**	**N_Male_**	**N_Nonbinary/Other_**
Reps. for Harris	89	NA	44.55 (14.78)	45	44	0
Reps. for Trump	340	NA	44.96 (14.05)	174	166	0
Democrats	294	NA	38.67 (11.81)	145	144	5
**Time 2—1 wk pre**						
	**N_Total_**	**Attrition**	**Age**	**Gender**		
			**M (SD)**	**N_Female_**	**N_Male_**	**N_Nonbinary/Other_**
Reps. for Harris	51	42.70%	45.61 (12.32)	25	26	0
Reps. for Trump	257	24.41%	45.77 (14.39)	125	132	0
Democrats	181	38.44%	38.79 (11.50)	94	83	4
**Time 3—2 wks post**						
	**N_Total_**	**Attrition**	**Age**	**Gender**		
			**M (SD)**	**N_Female_**	**N_Male_**	**N_Nonbinary/Other_**
Reps. for Harris	43	15.69%	47.67 (14.57)	19	24	0
Reps. for Trump	244	5.06%	46.32 (14.66)	117	123	0
Democrats	163	9.94%	32.29 (11.15)	84	73	3

Note. Missing data on gender are missing for four Reps. for Trump and three Democrats at the third time point.

**Table 2:** Standardized Effect Sizes for Predictors of Warmth Toward RHs and RTs Among Each Sample (RTs, RHs, Democrats).

Predictor	Warmth Toward RHs			Warmth Toward RTs		
	RTs	RHs	Dems	RTs	RHs	Dems
Across Time Points						
Intercept	−52.28 ***	61.96 ***	40.30 ***	78.01 ***	−46.75 ***	−80.90 ***
Perceived Loyalty to Rep. Party	10.61 ***	8.53 **	9.95 ***	6.37 ***	19.86 ***	8.04 ***
Perceived Norm Priority (Country Over Party)	−0.32	−0.49	2.88 *	−0.16	2.90	0.98
Perceived Likelihood of Supporting Rep. Party	6.29 ***	1.31	−2.36	0.65	5.10	−1.29
Perceived Likelihood of Dissenting from Rep. Party	−0.89	3.15	7.05 ***	2.14 *	7.73	6.14 ***
Perceived Republican-ness	18.39 ***	5.06 *	3.68 *	2.92 ***	9.07*	0.87
Perceived Democratic-ness	1.74	5.22 *	5.04 **	2.36 **	6.67	6.69 ***
Identification with Republicans	0.64	1.03	2.78	11.05 ***	8.50*	6.31 ***
Identification with Democrats	12.10 ***	7.03 *	0.05 ***	−2.11 *	−2.34	−3.13 **
6 Weeks Before the 2024 Presidential Election						
Intercept	−43.38 ***	49.42 ***	23.24 ***	73.31 ***	−48.38 ***	−80.61 ***
Perceived Loyalty to Rep. Party	7.11 *	6.30	12.33 ***	8.30 ***	20.17 **	9.85 ***
Perceived Norm Priority (Country Over Party)	13.18 ***	16.15 **	21.70 ***	5.09 *	5.44	1.34
Perceived Likelihood of Supporting Rep. Party	3.88	−6.78	−1.87	−0.61	7.22	−2.32
Perceived Likelihood of Dissenting from Rep. Party	−1.26	2.46	5.70 *	4.37 *	14.66 *	5.92 *
Perceived Republican-ness	20.09 ***	6.03	3.33	4.63 ***	8.39	0.21
Perceived Democratic-ness	5.21 *	5.14	8.68 ***	2.72 *	4.38	9.02 ***
Identification with Republicans	3.21	7.22	5.89 *	12.81 ***	6.18	5.10 *
Identification with Democrats	9.89 ***	10.91 *	9.34 ***	−3.08 *	−9.88	−4.72 **
1 Week Before the 2024 Presidential Election						
Intercept	−49.6 ***	46.46 ***	38.96 ***	73.58 ***	−44.76 ***	−82.02 ***
Perceived Loyalty to Rep. Party	8.00 *	0.65	9.78 *	9.33 ***	25.41 **	7.56 **
Perceived Norm Priority (Country Over Party)	−9.81 *	−20.72 *	−2.05	−7.70**	−4.54	3.83
Perceived Likelihood of Supporting Rep. Party	7.36 *	10.73	−1.57	−3.36	−2.04	−1.50
Perceived Likelihood of Dissenting from Rep. Party	7.39	7.50	9.68 **	3.49	−6.89	6.95 ***
Perceived Republican-ness	15.70 ***	10.95	3.33	4.45 **	4.59	2.13
Perceived Democratic-ness	−3.52	4.12	7.42 *	3.37 *	18.96 *	10.02 ***
Identification with Republicans	3.46	−0.77	4.65	11.91 ***	12.78 *	9.32 ***
Identification with Democrats	22.04 ***	5.91	11.57 ***	−1.12	11.47	−3.06
2 Weeks After the 2024 Presidential Election						
Intercept	−52.24 ***	61.99***	40.37 ***	76.21 ***	−24.94 **	−79.13 ***
Perceived Loyalty to Rep. Party	11.52 **	13.46	15.82 **	4.51 *	23.76	8.46 **
Perceived Norm Priority (Country Over Party)	−2.40	3.41	5.31	−4.34	−7.97	−2.47
Perceived Likelihood of Supporting Rep. Party	1.70	−5.90	−7.90	0.53	6.49	−2.43
Perceived Likelihood of Dissenting from Rep. Party	−2.47	−5.88	3.03	4.03*	3.90	5.23
Perceived Republican-ness	22.69 ***	9.55	5.79	7.81 ***	8.52	0.87
Perceived Democratic-ness	1.29	5.12	4.28	2.14	5.06	9.76 **
Identification with Republicans	−0.13	−3.14	12.87 ***	11.93 ***	11.89	10.27 ***
Identification with Democrats	12.01 ***	6.23	16.38 ***	−0.38	−0.45	−4.71 *

Note. Standardized effect sizes represent coefficients from separate regression models predicting warmth toward Republicans for Harris (RHs; first three columns) and Republicans for Trump (RTs; last three columns). Across and within individual time points, columns represent distinct models for each participant group (RTs, RHs, and Democrats). *** *p* < 0.001. ** *p* < 0.01. * *p* < 0.05.

## Data Availability

All preregistrations, materials, data, analysis scripts, and Supplementary Materials for the current studies are available on OSF: https://osf.io/7t9mw/?view_only=e2c602b8fe514d69be6e01428f855978 (accessed on 7 January 2026).

## Note

1 After the election, 96.31% of RTs, 86.05% of RHs, and 91.41% of Democrats indicated that they voted for their supported candidate.

## Supplementary Materials

The following supporting information can be downloaded at https://www.mdpi.com/article/10.3390/bs16010126/s1, Figure S1: Warmth Toward Republican and Democrat Candidates by Sample, ANES 2024; Figure S2: Identification with Republicans and Democrats Across Time; Table S1: Deviations from the Preregistrations; Table S2: Exploratory Factor Analysis on Perception Items at Time 1; Table S3: Mean Political Perceptions and Warmth Toward Republicans for Harris; Table S4: Shifts in Political Perceptions and Warmth Toward Deviants Around the Election; Table S5: Correlations Between Self-Perception Outcomes at Time 3 (2 wks post election) (RHs).
