# Supplementary material for: Redefining Loyalty: How Political Deviants Maintain Positive Self-Views Amid Ingroup Rejection
Source: Behav Sci (Basel). 2026 Jan 16;16(1):126. doi: 10.3390/bs16010126 (PMC12837132; doi:10.3390/bs16010126)
Supplement: Supplementary file 1 [file behavsci-16-00126-s001.zip › behavsci-4015187-supplementary.pdf]

Allies or Outsiders? Republicans for Harris in the 2024 Presidential Election

Supplementary Materials

Table S1.

Deviations from the Preregistrations

Aspect of Study	Preregistered Plan	Actual Implementation	Reason for Deviation
Analyses	Proposed testing mediation models exploring whether perceived likelihood of support, likelihood of dissent and having friends who are outgroup members explains negative attitudes toward RHs.	Reported correlations between outcome variables instead of conducting a mediation.	Results of the preregistered exploratory factor analysis led to collapsing having friends who are outgroup members into the likelihood of dissent variable. Theoretical and empirical support for a mediation was deemed insufficient, so a mediation analysis was not conducted.
Data Collection Timeline	Proposed collecting the third wave of data 7 days after the election	Collected the third wave of data 14 days after the election	Due to delays in IRB approval, data collection for time point 3 was delayed

Table S2

Exploratory Factor Analysis on Perception Items at Time 1

Item	Loading Reps for Harris Targets)	Loading (Reps for Trump Targets)
Loyalty to Republican Party	α = .882	α = .904
…are traitors (Reversed)	0.89	0.92
…have betrayed their party (Reversed)	0.95	0.91
…are TRUE Republicans	0.57	0.73
Likelihood of Supporting Republican Party	α = .828	α = .763
…are likely to vote in favor of conservative policies in the future	0.69	0.74
…likely have many friends who are Republican	0.82	0.66
…are likely to support Republican candidates in other elections	0.84	0.68
Likelihood of Dissent from Republican Party	α = .841	α = .843
…are likely to vote in favor of liberal policies in the future	0.70	0.76
…likely have many friends who are Democrat	0.65	0.57
…are likely to support Democratic candidates in other elections	0.84	0.87
…are likely to become Democrat in the future	0.68	0.86

Note. An exploratory factor analysis (EFA) with a promax rotation was conducted to find the minimum residual solution. Based on a scree plot, we determined that a three-factor model would best explain the data. We find similar results using a varimax rotation, assuming an orthogonal rotation, reported in the Supplementary Materials.

ANES Data

Data come from the American National Election Studies (ANES) 2024 Time Series Study, a nationally representative survey of 5521 U.S. adults. The study uses a two-wave panel design, consisting of a pre-election interview fielded from September through Election Day (November 5, 2024) and a post-election re-interview fielded from November 6, 2024 through January 2025. Respondents were recruited using probability-based sampling and surveyed primarily online, with follow-up interviews administered to recontacted participants after the election.

Using reported candidate preferences and party registration, we identified 966 Democrats who voted for Harris (DHs), 665 Republicans who voted for Trump (RTs), 100 Republicans who voted for Harris (RHs), and 91 Democrats who voted for Trump (DTs). Attitudes toward the 2024 Democratic and Republican Presidential Candidates were assessed using feeling thermometers. We examined group differences using a multilevel models with random intercepts for time point and respondent, and extracted pairwise comparisons using emmeans.

Across both waves, DHs reported significantly warmer feelings toward Harris than RHs (Pre: b = 14.9***, Post: b = 16.8***), DTs (Pre: b = 40.5***, Post: 54.5***) and RTs (Pre: b = 62.6***, Post: 66.8***); RHs reported warmer feelings toward Harris than DTs (Pre: b = 25.6***, Post: 37.7***) and RTs (Pre: b = 47.7***, Post: 50.0***); and DTs reported warmer feelings toward Harris than RTs (Pre: b = 22.1***, Post: 12.3***).

Additionally, RTs reported significantly warmer feelings toward Trump than DTs (Pre: b = 12.95***, Post: 12.64***), RHs (Pre: 45.94***, Post: 63.67***) and DHs (Pre: 61.55***, Post: 69.87***); DTs reported warmer feelings toward Trump than RHs (Pre: 32.99***, Post:

51.03***) and DHs (Pre: 48.60***, Post: 57.24***); and RHs reported warmer feelings toward Trump than DHs (Pre: 15.61***, Post: 6.21*).

Figure S1.

Warmth Toward Republican and Democrat Candidates by Sample, ANES 2024

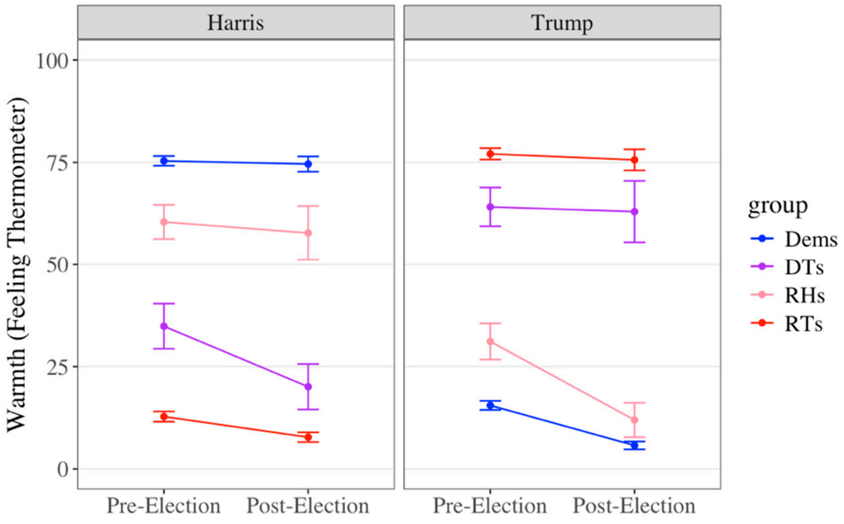

Examining change over time, supporters of Harris reported less warmth toward Trump after the election than before (DHs: b = -9.79***; RHs: b = -19.19***). Similarly, supporters of Trump reported less warmth toward Harris after the election compared to before (RTs: b = -5.04***; DTs: b = -14.79***). By contrast, warmth toward one's supported candidate remained stable across waves, suggesting that the election results primarily influenced outparty antipathy rather than inparty affection.

Together, these nationally representative ANES results complement our study by showing that political deviants, both Republicans for Harris and Democrats for Trump, occupied an affective middle group in evaluations of partisan leaders. Whereas our study focused on feelings of warmth toward mainstream and dissident party members and the factors shaping

these evaluations, the ANES data demonstrate that this distinctive positioning is also visible in large-scale patterns of candidate evaluation. Furthermore, the ANES results extend our findings by including Democrats for Trump, suggesting that partisan deviants on both sides of the aisle show reduced inparty warmth and outparty antipathy.

Data Source: American National Election Studies. 2025. ANES 2024 Time Series Study Full Release [dataset and documentation]. August 8, 2025 version. www.electionstudies.org

Identity Certainty. At the third time point (2 wks post) only, Republicans also rated how certain they are in their political identity on a 6-pt scale from "Not at all certain" to "Completely certain."

Identity Centrality. The centrality of Republicans' party identity ($r = 0.59$, $p < .001$) was measured with two items: "My identity as a Republican is more important to me than my identity as someone who supports [Trump/Harris]" and "I am a Republican first, and a supporter of [Trump/Harris] second".

Identity Security. We also aimed to assess how secure participants felt in their Republican identities despite their deviance, which we refer to as identity security. The identity security of Republicans for Harris ($r = 0.43$**) was measured with two items: "I can be someone who supports Harris without feeling disconnected from the Republican Party" and "When thinking about my political identity, my support for Harris is central to who I am". Identity centrality and identity security were assessed on 7-pt Likert scales from "Strongly disagree" to "Strongly agree."

Ingroup (RTs) vs. Outgroup (Democrats) perceptions of and warmth toward RHs

As shown in Table 3, Democrats perceived RHs as more loyal, *b* = 2.51, *t*(420) = 25.32, *p* < .001, 95% CI [2.27, 2.74], higher in Republican-ness, *b* = 40.31, *t*(399) = -18.85, *p* < .001, 95% CI [35.30 45.33], and lower in Democrat-ness, *b* = -22.13, *t*(395) = -9.14, *p* < .001, 95% CI [-27.80, -16.43], than RTs perceived RHs to be. Additionally, Democrats felt warmer toward RHs than RTs did, *b* = 92.90, *t*(414) = 20.04, *p* < .001, 95% CI [82.00, 103.80]. While Democrats' and RT's warmth toward RHs did not significantly shift between time points, follow-up analyses revealed a significant temporal effect: the greater number of days that had passed since Election Day (November 5) when participants completed the post-election survey, the warmer RTs' attitudes toward RHs, *b* = 4.59, *t*(220) = 2.73, *p* = .007, 95% CI [1.28, 7.90], and the colder Democrats' attitudes toward RHs, *b* = -3.69, *t*(123) = -2.18, *p* = .031, 95% CI [-7.04, -0.34]. These results suggest that as the salience of the threat posed by ingroup deviance diminished over time, ingroup members began to view deviants more positively, while outgroup members, who could no longer benefit as much from the deviance, began to view them more negatively.

Table S3

Mean Political Perceptions and Warmth Toward Republicans for Harris

**Outcome**	**Sample**	**M (SD)**
Loyalty to Republican Party	RT	2.42 (1.15)
	Dem	5.04 (0.80)[a]
	RH	5.06 (0.80)[a]
Likelihood of Supporting Republican Party	RT	3.03 (1.18)
	Dem	4.62 (0.87)[a]
	RH	4.46 (0.87)[a]

Likelihood of Dissenting from Republican Party	RT	4.58 (0.98)
	Dem	3.24 (0.88)[a]
	RH	3.45 (0.84)[a]
Republican-ness	RT	21.41 (23.70)
	Dem	62.42 (25.87)[a]
	RH	62.92 (25.72)[a]
Democrat-ness	RT	61.47 (23.70)
	Dem	38.92 (24.42)[a]
	RH	46.62 (27.88)[a]
Warmth	RT	-56.35 (51.63)
	Dem	39.26 (44.55)
	RH	57.09 (39.18)

Note. Within each outcome, means that do not share a superscript differ significantly from one another at $p < .05$, based on pairwise comparisons using the "emmeans" package following LMEs with Sample entered as a fixed effect and Time and Participant entered as random effects. Exact comparisons are reported in the Supplementary Materials.

(Mis)alignment between political perceptions of deviants and their self-perceptions

Results reveal large gaps between how the majority ingroup subset (RTs) think about RHs and how RHs think about themselves. RHs viewed themselves as more loyal than RTs perceived them to be, $b = 2.25$, $t(686) = 16.07$, $p < .001$, 95% CI [1.92, 2.58], as well as less likely to dissent, $b = -1.06$, $t(617) = -8.27$, $p < .001$, 95% CI [-1.36, -0.76], and more likely to support the Republican party, $b = 1.26$, $t(590) = 8.68$, $p < .001$, 95% CI [0.92, 1.60]. Similarly,

RHs viewed themselves as lower in Democrat-ness than RTs perceived them to be, *b* = -14.60, t(493) = -3.92, p < .001, 95% CI [-23.30, -5.85], and higher in Republican-ness, *b* = 40.04, t(526) = 12.38, p < .001, 95% CI [32.40, 47.65].

By comparison, Democrats' perceptions of RHs were not significantly different from RHs' self-perceptions. There were no significant differences in Democrats' perceptions and RHs self-perceptions of Republican loyalty, *b* = -0.26, t(656) = -1.72, p = .199, 95% CI [-0.61, 0.09], likelihood of dissent, *b* = -0.27, t(595) = -1.98, p = .119, 95% CI [-0.59, 0.05], likelihood of supporting the Republican party, *b* = -0.30, t(571) = -1.95, p = .126, 95% CI [-0.06, 0.66]. Democrat-ness, *b* = -7.53, t(485) = -1.92, p = .135, 95% CI [-16.76, 1.70], and Republican-ness, *b* = 0.27, t(514) = 0.08, p = .997, 95% CI [-7.77, 8.30].

Ingroup (RTs) perceptions of and warmth toward RHs vs. RTs vs. Democrats

In line with prior work (Abrams et al., 2005), we find RTs viewed RHs more negatively compared to other Republicans. Across time points, RTs saw RHs as less loyal than RTs, *b* = -2.85, *t*(1531) = -57.94, *p* < .001, 95% CI [-2.96, -2.73], as well as less likely to support the Republican party, *b* = -2.20, *t*(1742) = -45.33, *p* < .001, 95% CI [-2.33, -2.08], and more likely to dissent from the Republican party, *b* = 2.42, *t*(1740) = 49.02, *p* < .001, 95% CI [2.29, 2.54]. RTs saw RHs as lower in Republican-ness than RTs, *b* = -71.38, *t*(2183) = -77.31, *p* < .001, 95% CI [-73.75, -66.69], and higher than Democrats, *b* = 13.23, *t*(2183) = 14.33, *p* < .001, 95% CI [10.85, 15.60]. Additionally, RTs saw RHs as higher in Democrat-ness than RTs, *b* = 40.32, *t*(2185) = 22.97, *p* < .001, 95% CI [35.81, 44.83], and lower than Democrats, *b* = -20.76, *t*(2185) = -11.83, *p* < .001, 95% CI [-25.28, -16.25]. Finally, RTs felt much less warm toward RHs than they did toward RTs, *b* = -134.37, *t*(1751) = -64.48, *p* < .001, 95% CI [-129.48, -

64.48], but no less warm than they feel toward Democrats, *b* = -2.85, *t*(1751) = -1.37, *p* = .358, 95% CI [-7.74, 2.04].

RT's perceptions of RHs remained stable across time points, with one exception: RTs perceived RHs as more likely to dissent at leading up to the election (between the first and second time points), *b* = 0.20, t(435) = 2.90, p = .011, 95% CI [0.04, 0.35]. This could reflect heightened deviance concerns immediately preceding the election. RTs' perceptions of RHs' likelihood of dissent did not significantly differ after the election. Overall, the consistency of RT's perceptions of RHs in the current study suggests that these perceptions were robust to any perceived enhancements to ingroup status following the election victory up to at least two weeks after the election.

Outgroup (Democrats) perceptions of and warmth toward RHs vs. RTs vs. Democrats

On the contrary, Democrats viewed RHs more positively than they viewed other Republicans. Across time points, Democrats saw RHs as more loyal, *b* = 2.48, *t*(869) = 33.85, *p* < .001, 95% CI [2.31, 2.65], less likely to support the Republican party, *b* = -0.79, *t*(420) = -14.29, *p* < .001, 95% CI [-0.93, -0.66], and more likely to dissent from the Republican party, *b* = 1.71, *t*(744) = 34.77, *p* < .001, 95% CI [1.60, 1.83], than they perceived RTs to be. Additionally, Democrats viewed RHs as more likely to support the Republican party, *b* = 1.56, *t*(407) = 15.85, *p* < .001, 95% CI [-1.79, -1.33], and less likely to dissent from the Republican party, *b* = -1.33, *t*(410) = 15.10, *p* < .001, 95% CI [-1.54, -1.22], than RTs viewed RHs to be. While Democrats saw RHs as lower in Republican-ness than RTs, *b* = -12.30, *t*(1244) = -28.96, *p* < .001, 95% CI [-16.86, -7.82], and higher than Democrats, *b* = 53.40, *t*(1244) = -30.40, *p* < .001, 95% CI [48.86, 57.89], they saw RHs as higher in Democrat-ness than RTs, *b* = 34.93, *t*(1243) = 30.84, *p*

< .001, 95% CI [32.01, 37.80], and lower than Democrats, *b* = -54.51, *t*(1243) = 48.14, *p* < .001, 95% CI [-57.40, -51.60]. Finally, Democrats felt less warm toward RHs than they did toward Democrats, *b* = -30.80, *t*(1000) = -13.28, *p* < .001, 95% CI [-36.0, -25.40], but warmer than they felt toward RTs, *b* = 120.70, *t*(1000) = 52.03, *p* < .001, 95% CI [115.20, 126.10]. Similar to those of RTs, Democrats' perceptions of RHs remained stable across time points, suggesting that their perceptions were robust to any perceived reductions in the utility of RHs following the election loss up to at least two weeks after the election.

RHs' perceptions of and warmth toward RHs vs. RTs vs. Democrats

RHs perceived themselves as more loyal Republicans, *b* = 2.09, t(248) = 15.64, p < .001, 95% CI [1.77, 2.40], more likely to dissent from the Republican party, *b* = 1.55, t(277.50) = 16.01, p < .001, 95% CI [1.30, 1.80], and less likely to support the Republican party, *b* = -0.81, t(248) = -7.90, p < .001, 95% CI [-1.08, -0.55], than they perceived RTs to be. Additionally, RHs viewed themselves as higher in Democrat-ness than they viewed RTs to be, *b* = 38.54, t(360) = 13.94, p < .001, 95% CI [31.40, 45.68], but lower than Democrats, *b* = -41.31, t(360) = -14.94, p < .001, 95% CI [-48.44, -34.20], and not significantly lower in Republican-ness than they viewed RTs to be, *b* = -8.58, t(361) = -2.47, p = .067, 95% CI [-17.55, 0.40], but higher than Democrats, *b* = 45.40, t(361) = 13.05, p < .001, 95% CI [36.42, 54.37]. Finally, RHs felt warmer toward their own group (RHs) than they felt toward Democrats, *b* = 28.90, t(286) = -4.91, p < .001, 95% CI [15.10, 42.80], and warmer toward Democrats than toward RTs, *b* = 71.30, t(286) = 12.11, p < .001, 95% CI [57.50, 85.20]. Together, these findings suggest RHs maintained strong identification with the Republican party and viewed themselves as highly loyal despite their support for Harris.

Further supporting this view, RHs scored above the midpoint in certainty about their political identities (M = 4.18, SD = 1.37) after the election, though they were less certain than RTs were, $b = -1.13$, $t(271) = -7.02$, $p < .001$, 95% CI [-1.44, -0.81]. RHs also scored above the midpoint in Republican centrality (i.e., how much they prioritized their party identity over their candidate preference) (M = 3.86, SD = 1.52), but considered their Republican identity to be less central than RTs did, b = -0.58, $t(270) = -2.15$, $p = .033$, 95% CI [-1.11, -0.05]. Finally, RHs scored above the midpoint on identity security (i.e., how secure they felt in their Republican identity despite their deviance) (M = 4.73, SD = 1.24). As shown in Table 4, RHs with higher identity centrality reported a lower likelihood of future dissent and higher likelihood of future support. In contrast, RHs with higher identity security reported a higher likelihood of future dissent. Additionally, RHs who saw themselves as more loyal Republicans reported a greater likelihood of future dissent, lower likelihood of future support, and saw themselves as both more Republican as well as more Democrat. These results provide support for Normative Conflict Model, suggesting that Republicans for Harris' loyalty to the Republican party may motivate their deviance from party norms.

Figure S2

Identification with Republicans (Left) and Democrats (Right) Across Time

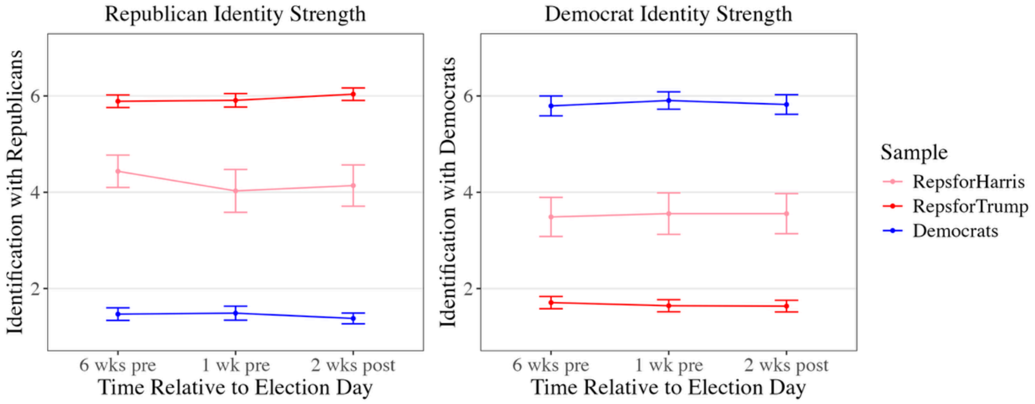

Note. Error bars represent 95% confidence intervals.

Table S4

Shifts in Political Perceptions and Warmth Toward Deviants Around the Election

Outcome	Time Period	b	t(df)	p	95% CI	
					Lower	Upper
**RTs' Perceptions of RHs**						
Republican Loyalty	T2 – T1	-0.06	-0.98(434)	.592	-0.20	0.08
	T3 – T2	0.10	1.57(434)	.258	-0.05	0.24
Likelihood of Dissent	T2 – T1	0.20	2.90(435)	.001	0.04	0.35
	T3 – T2	-0.07	-1.06(436)	.538	-0.23	0.09
Likelihood of Support	T2 – T1	-0.05	-0.68(435)	.772	-0.24	0.13
	T3 – T2	0.04	-0.55(436)	.846	-0.14	0.23
Democrat-ness	T2 – T1	-3.91	-1.49(437)	.296	-10.07	2.25
	T3 – T2	3.36	1.29(437)	.403	-2.78	9.50
Republican-ness	T2 – T1	-0.06	-0.04(435)	.999	-3.99	3.87
	T3 – T2	1.55	0.93(435)	.621	-2.36	5.47
Warmth	T2 – T1	-2.99	-1.00(435)	.579	-10.03	4.06
	T3 – T2	6.07	2.03(435)	.106	-0.95	13.09
**Democrats' Perceptions of RHs**						
Republican Loyalty	T2 – T1	-0.03	-0.53(245)	.859	-0.18	0.12
	T3 – T2	-0.03	-0.47(246)	.887	-0.18	0.12
Likelihood of Dissent	T2 – T1	0.03	0.46(245)	.890	-0.11	0.16
	T3 – T2	-0.03	-0.48(245)	.881	-0.17	0.11
Likelihood of Support	T2 – T1	0.10	1.46(246)	.311	-0.06	0.27
	T3 – T2	0.02	0.35(246)	.936	-0.14	0.19

Democrat-ness	T2 – T1	1.14	0.52(247)	.861	-4.02	6.31
	T3 – T2	1.04	0.47(248)	.886	-4.16	6.23
Republican-ness	T2 – T1	-0.15	0.06(248)	.998	-5.85	6.15
	T3 – T2	3.96	1.55(248	.270	-2.07	10.00
Warmth	T2 – T1	1.05	0.31(248)	.949	-7.01	9.10
	T3 – T2	-5.72	-1.67(248)	.219	-13.90	2.36
**RHs' Self-Perceptions**						
Republican Loyalty	T2 – T1	0.20	1.56(70.3)	.270	-0.10	0.50
	T3 – T2	0.10	0.78(68.6)	.714	-0.20	0.40
Likelihood of Dissent	T2 – T1	-0.07	0.60(69.2)	.821	-0.33	0.20
	T3 – T2	0.13	1.13(67.9)	.500	-0.14	0.39
Likelihood of Support	T2 – T1	-0.11	-0.78(70.6)	.718	-0.47	0.24
	T3 – T2	-0.04	-0.30(68.8)	.952	-0.40	0.31
Democrat-ness	T2 – T1	13.54	2.73(72)	.022	1.65	25.43
	T3 – T2	-3.70	-0.74(70)	.736	-15.73	8.33
Republican-ness	T2 – T1	4.29	0.92(72.1)	.627	-6.82	15.40
	T3 – T2	2.36	0.50(70)	.870	-8.88	13.60
Warmth	T2 – T1	8.34	1.45(69.8)	.324	-5.49	22.20
	T3 – T2	0.90	0.15(68.3)	.987	-13.03	14.80
Republican Identity Strength	T2 – T1	-0.48	-2.88(70.2)	.015	-0.88	-0.08
	T3 – T2	0.18	1.10(68.8)	.521	-0.22	0.58
Democrat Identity Strength	T2 – T1	0.11	0.57(70.8)	.838	-0.35	0.57
	T3 – T2	0.01	0.06(69.1)	.998	-0.45	0.48
**RHs' Perceptions of RTs**						

Republican Loyalty	T2 – T1	-0.40	-2.12(69.5)	.094	0.05	-0.85
	T3 – T2	0.14	0.72(68.1)	.750	-0.32	0.59
Likelihood of Dissent	T2 – T1	-0.06	-0.38(70.7)	.925	-0.42	0.31
	T3 – T2	0.10	0.65(68.9)	.792	-0.27	0.47
Likelihood of Support	T2 – T1	-0.01	-0.06(70.5)	.998	-0.36	0.34
	T3 – T2	0.20	1.33(68.7)	.385	-0.16	0.55
Democrat-ness	T2 – T1	-1.13	-0.27(73.7)	.962	-11.28	9.03
	T3 – T2	3.79	0.88(71.6)	.656	-6.54	14.12
Republican-ness	T2 – T1	-8.93	-1.40(72.8)	.345	-24.2	6.30
	T3 – T2	-0.42	-0.07(70.6)	.998	-15.90	15.03
Warmth	T2 – T1	-10.30	-1.11(70.6)	.509	-32.45	11.80
	T3 – T2	25.50	2.73(68.8)	.022	2.73	3.12
**RHs' Perceptions of Democrats**						
Democrat-ness	T2 – T1	-2.54	-0.68(74.6)	.778	-11.50	6.44
	T3 – T2	-1.03	-0.27(72.7)	.269	-10.20	8.13
Republican-ness	T2 – T1	10.58	2.29(73)	.064	-0.49	21.64
	T3 – T2	-7.10	-1.51(70.8)	.291	-18.33	4.13
Warmth	T2 – T1	-7.17	-1.07(69.5)	.536	-23.20	8.89
	T3 – T2	-0.40	-0.06(68.1)	.998	-16.60	15.77

Shifts in deviants' political perceptions and warmth toward themselves and others around the election

Leading up to the election (between the first and second time points), RHs viewed themselves as higher in Democrat-ness, $b = 13.54$, $t(72.00) = 2.73$, $p = .022$, 95% CI [1.65,

25.43], and identified less strongly with Republicans, *b* = -0.48, t(70.20) = -2.88, p =.015, 95% CI [-0.88, -0.08]. No other significant differences in RHs' self-perceptions were observed during the pre-election period. After the election (at the third time point), RHs viewed themselves as marginally more loyal to the Republican party compared to the first time point, *b* = 0.29, *t*(70.00) = 2.37, *p* = .053, 95% CI [-0.003, 0.59]. No other significant differences in RHs' self-perceptions emerged during the post-election period.

Across time, there were no significant changes in RHs' political perceptions of RTs and Democrats. Additionally, RHs' warmth toward Democrats remained stable, however their warmth toward RTs significantly increased after the election, *b* = 25.50, *t*(68.80) = 2.73, *p* = .022, 95% CI [2.73, 3.12].

These findings may reflect a dynamic pattern in RHs' political identity alignment around the election. Leading up to the election, RHs appeared to align more closely with Democrats. Following the Republican presidential victory, RHs align more closely with Republicans, potentially reflecting a move toward partisan unity after the election.

Table S5

Correlations between Self-Perception Outcomes at Time 3 (2 wks post election) (RHs)

	**Loyalty**	**Perceived Norm Priority**	**Support**	**Dissent**	**Republican-ness**	**Democrat-ness**	**Rep Identification**	**Dem Identification**
Perceived Norm Priority	0.67***							
Support	-0.27**	-0.39***						
Dissent	0.43***	0.66***	-0.48***					
Republican-ness	0.34***	0.06	0.16	-0.07				
Democrat-ness	0.46***	0.48***	-0.46***	0.65***	-0.41***			
Rep Identification	0.06	0.00	0.28***	-0.12	0.07	-0.02		
Dem Identification	-0.13	0.01	-0.32***	0.35***	0.03	0.21**	-0.16*	
Warmth	0.77***	0.62***	-0.35*	0.47***	0.12	0.45***	0.02	0.07

Note. ***p < .001, **p < .01, *p < .0
